# A Method of Preventing or Diminishing Pain, in Several Operations of Surgery

**Published:** 1784

**Authors:** 


III. *A Method of preventing or diminishing Pain, in several Operations of Surgery. By* James Moore, *Member of the Surgeons Company of London.* 8vo. Cadell, *London,* 1784. 50 pages, with a copper-plate.

IT is very properly observed by the ingenious writer of the work now before us, that the most essential improvements that can be made in surgery, are unquestionably those which render operations safer, and diminish the danger of the patient's life. But what can diminish the acuteness of the pain without increasing the danger, is also an improvement very much to be wished.

The common uneasy sensation which is included in the general term of pain, is indeed of little consequence. But when people confider the degree of pain given by fome furgical operations, they muft acknowledge, that to diminifh or prevent a few minutes of *fuch* pain, is an object highly defirable, both to the patient and furgeon. Reflections of this kind ftruck our author, it feems, very early after he began to ftudy his profeffion, and he made various experiments without fuccefs, in fearch of fomething which might mitigate the violence of the pain in furgical operations. Of late he refumed the fubject, and reflecting on the nature of the nerves, and on fome facts concerning them, he was led to an idea, which he flatters himfelf, will go a confiderable way to the accomplifhing the object he had in view.

The firft thought that occurred to him was, that, to cut the trunk of a nerve going to a limb, might be done with little pain, and enable us to perform the amputation with no pain at all. But a very little reflection convinced him that this was impracticable.

He then thought his end might poffibly be accomplifhed by compreffion; and was encouraged in this idea, by having often felt that fenfation, which proceeds from compreffing the fciatic nerve by fitting in a particular pofition.

It

It occurred to him at the ſame time, that the compreſſion of the nerve might be made moſt effectually by means of a tourniquet.

Of the experiments Mr. Moore firſt made for this purpoſe, on his own perſon, the reſult was not ſuch as he expected; a ſtrong compreſſion of the ſciatic nerve, juſt as it paſſes over the lower edge of the iſchium, having failed to deprive his leg and foot of their ſenſibility. This failure, as he afterwards diſcovered, was owing to the preſſure not being continued during a ſufficient length of time; for in a ſubſequent experiment, when the tourniquet remained on fourteen minutes, his foot was quite numbed; and in half an hour his foot, leg, and the outſide of his thigh, were ſo perfectly inſenſible, that when he pricked them with pins, he felt nothing. Still, however, part of the inſide of his thigh and leg retained ſome degree of feeling. This he aſcribed to his not having compreſſed the crural and obturator nerves. He now ſlackened the tourni quet, and in a few minutes ſenſation, and the power of motion, returned to his limb.

He now cauſed a bandage to be made with two thick compreſſes, one of which was placed on the crural and obturator nerves, and the

 other other on the ſciatic, at the upper part of the thigh. A tourniquet was applied and tightened, and in half an hour he had not the leaſt feeling, upon pricking any part of his limb.

Mr. Moore acknowledges, that an uneaſy ſenſation is produced by the compreſſion, but how infinitely inferior this is to the pain of amputations, he thinks may be conceived from his having borne it eaſily for ſo long a period.

As the total ſtoppage of the circulation for ſo long a time as is neceſſary to render the limb completely inſenſible, may be conſidered as an objection to the uſe of a tourniquet, he has contrived an inſtrument, formed of a curved piece of iron, covered with leather, and of ſufficient capacity to contain the thigh within its curve. At one end of this inſtrument, is a firm compreſs of leather, which is to be placed on the ſciatic nerve. A ſcrew paſſes through a tube at the other extremity of the inſtrument, and terminates in an oval compreſs, which is to be placed on the crural nerve. By this inſtrument the compreſſion is confined to two points, which are nearly oppoſite to each other. All the reſt of the limb is left free and uncompreſſed. An engraving is given of the compreſſor applied to the thigh, and likewiſe of of a ſmaller one ſuited to the arm. The latter is directed to be applied to the plexus in the axilla, cloſe to the humeral artery.

Mr. Moore very candidly allows, that in amputations of the lower limbs above the knee, the compreſſor will not be able to diminiſh the pain in ſuch a degree, as when the operation is to be performed below the knee; which is owing, he obſerves, to ſome branches of the lumbar nerves, to the obturator nerve, and to branches which the ſciatic and crural nerves ſend off before they reach the thigh, not being compreſſed by the inſtrument. This defect, he thinks, may be ſupplied by applying a common tourniquet, and keeping it tight for fifteen or twenty minutes before the operation.

Our author gives a very accurate account of a caſe of amputation below the knee, in St. George's hoſpital, in which, by the favour of Mr. Hunter, he had an opportunity of trying the effect of his inſtrument. This experiment, which had all the ſucceſs he expected, though far from being deciſive, will, he thinks, be ſufficient to excite a very thorough inveſtigation of the ſubject.

It ſeems right to obſerve, however, that in this caſe a grain of opium was given to the patient tient about a quarter of an hour before the operation, with a view, as we are told, to diminiſh the ſmarting of the wound after it.—Much ſtreſs, perhaps, cannot be laid on ſo ſmall a doſe of opium, even by thoſe who may be moſt diſpoſed to doubt of the efficacy of the author's invention; but the experiment would certainly have been more ſatisfactory without it.

In the courſe of his pamphlet, Mr. Moore points out the advantages that may be expected to reſult from his invention, and attempts to obviate ſuch objections as he thinks may be brought againſt it; but for theſe, and other particulars, we muſt refer our readers to the work itſelf.

